# Older Adult Substance Use, Support, Health, and Well-Being: Understanding a Complex Picture

**DOI:** 10.1093/geroni/igab046.1112

**Published:** 2021-12-17

**Authors:** Bethany Bareham, Verena Cimarolli, Alexis Kuerbis

## Abstract

Substance Use Disorders (SUDs), particularly hazardous alcohol use and Opioid Use Disorder (OUD), are increasingly common in older populations. Both are associated with poor mental and physical wellbeing. Alcohol use can also contribute positively to older people’s social and emotional wellbeing, and may be used by older people in managing pain. Health concerns can make older people more aware of the impact of their lifestyle, including substance use, for their health. The relationship between substance use, health and wellbeing in old age is complex, raising questions for how older people with SUDs are best supported. We aim to give insight into this complexity, drawing on five recent studies. Our first presentation examines the impact of health concerns in old age on alcohol use. Our second presentation explores the complicated relationship between experiences of pain, and alcohol use with age. Our third presentation considers wellbeing in older people with OUD, and the impact of Adverse Childhood Experiences, psychosocial, health and sociodemographic factors; and what this means for how wellbeing is promoted in this population. Our fourth presentation explores how those supporting people with dementia navigate alcohol use amongst older residents, in a care context where autonomy is prioritised, but health risk behaviours must be managed. Our final presentation considers alcohol use in ‘the new normal’; examining the impact of the COVID-19 pandemic on older people’s alcohol use, and motivations to engage in healthier/unhealthy use. We consider implications of these complex considerations around addressing substance use amongst older people for practice.

